# Supplementary material for: CRISPR-Cas12a exhibits metal-dependent specificity switching
Source: Nucleic Acids Res. 2024 Jul 18;52(16):9343–59. doi: 10.1093/nar/gkae613 (PMC11381342; doi:10.1093/nar/gkae613)
Supplement: gkae613_Supplemental_Files [file gkae613_supplemental_files.zip › Cas12a_metal_manuscript_SI.pdf]

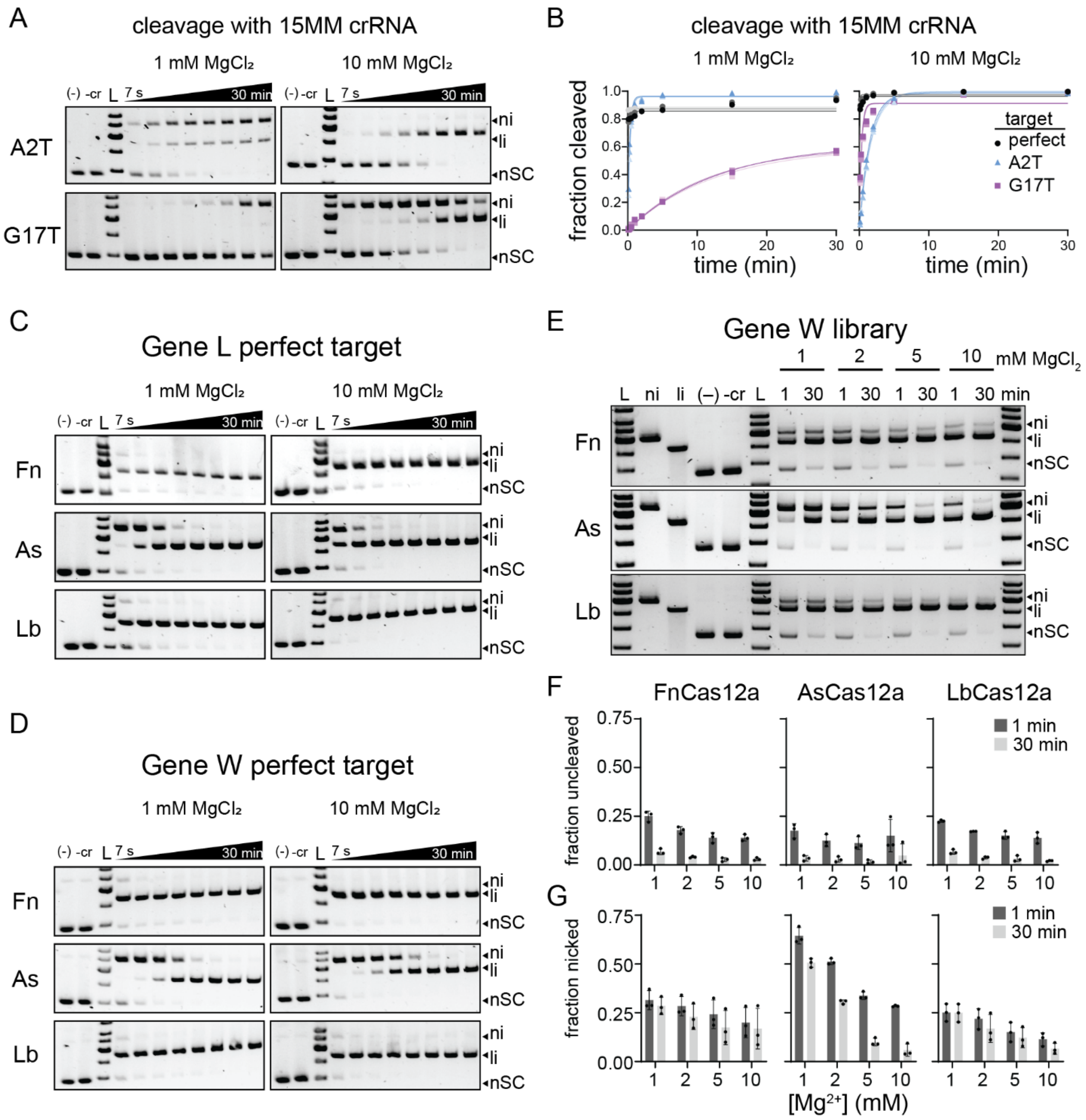

Figure S1: Cleavage of gene L and W targets and gene W target library (Related to Figure 1)

A-B) Cleavage and quantification of mutant gene L targets by FnCas12a bearing a crRNA containing a mismatch at position 15. For agarose gels in (A), the time points are 7 s, 15 s, 30 s, 1 min, 2 min, 5 min, 15 min, and 30 min. ni = nicked, li = linear, nSC = negatively supercoiled. (–) lane contains no protein, -cr contains protein but no crRNA. Both controls contained the indicated $MgCl_2$ concentration and were incubated at 37 °C for 30 min. Each gel is

representative of three replicates. The triplicate gels are quantified in (B) and fit to a single-exponential rate equation to derive the rate constants reported in Fig. 1C. Cleavage of the perfect gene L target cleaved by FnCas12a bearing the mismatched crRNA is also shown for comparison.

C-D) Cleavage of the perfectly matched gene L (C) and gene W (D) targets by each Cas12a ortholog at 1 and 10 mM $MgCl_2$. Timepoints and labels are as in (A). Both controls contained 10 mM $MgCl_2$ and were incubated at 37 °C for 30 min. Gels are representative of three replicates. For FnCas12a cleaving gene L, the same gels are shown in Fig. S5A.

E) Gene W plasmid library cleavage at four Mg^{2+} concentrations for FnCas12a (Fn), AsCas12a (As), and LbCas12a (Lb). ni = nicked, li = linear, nSC = negatively supercoiled. (–) lane contains no protein, -cr contains protein but no crRNA. Both controls contained 10 mM $MgCl_2$ and were incubated at 37 °C for 30 min. Gel is representative of three replicates.

F-G) Quantification of fraction uncleaved (F) or nicked (G) for the gene W library. The average of three replicates is plotted, with individual data points shown as dots and error bars representing standard deviation.

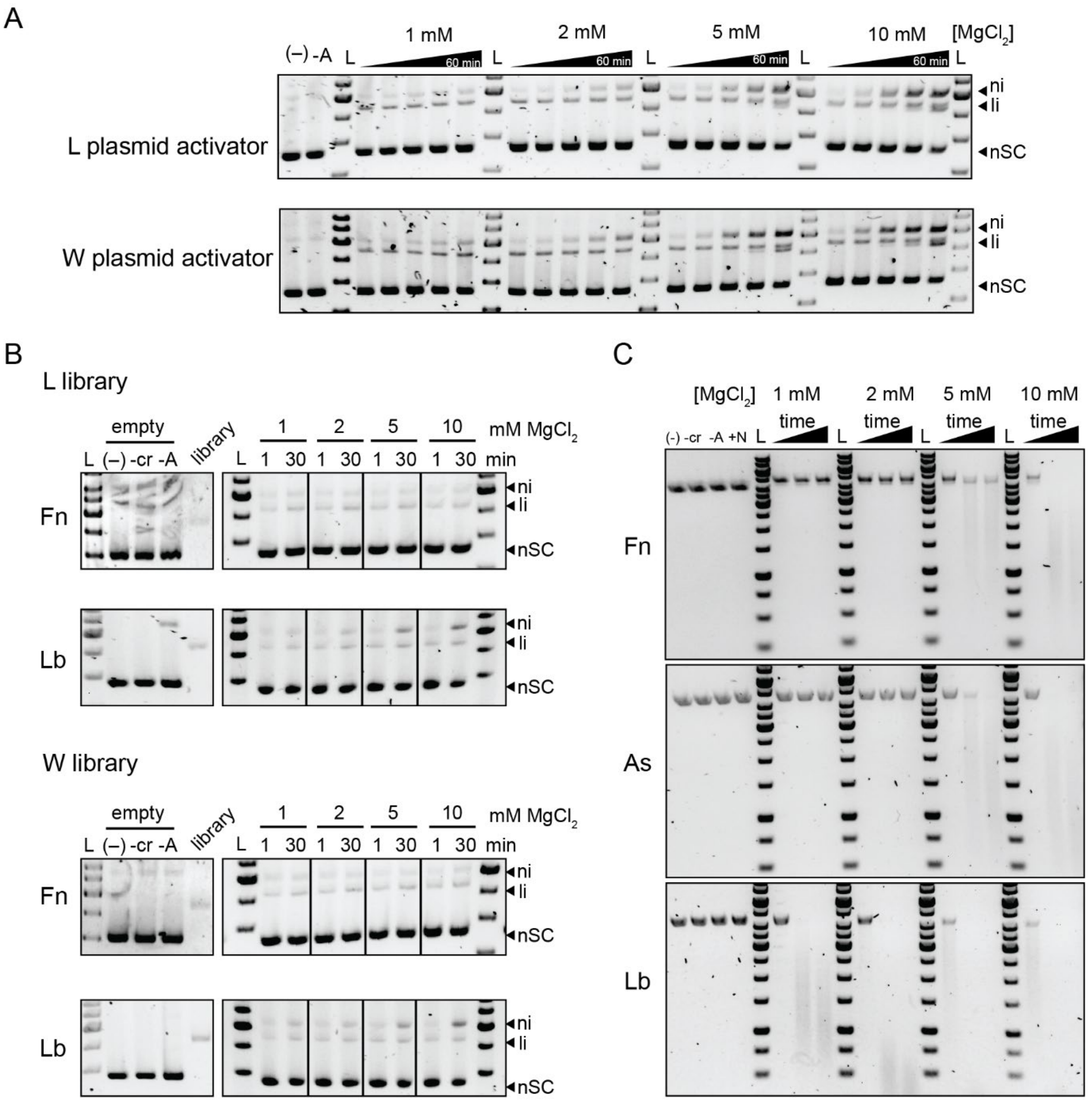

Figure S2: Collateral cleavage by Cas12a is Mg^{2+}-dependent (Related to Figure 1)

A) Cleavage of an empty pUC19 plasmid that lacks any complementarity to the crRNA by LbCas12a activated with either a gene L and gene W target pUC19 plasmid. The empty plasmid was in 10X excess of the activator target plasmid. The target plasmid was linearized by the first time point, and additional cleavage products observed at subsequent time points are most likely due to cleavage of the empty pUC19 plasmid. Time points: 1 min, 5 min, 15 min, 30 min, 60 min. The first two lanes are controls with empty pUC19 plasmid without any protein added (–)

or with Cas12a-crRNA but without the activator target plasmid added (-A) at 10 mM $MgCl_2$ incubated at 37 °C for the longest time point.

B) Cleavage of an empty pUC19 plasmid that lacks any complementarity to the crRNA by Fn or LbCas12a activated with either the gene L and gene W target plasmid library. The empty plasmid was in 10X excess of the plasmid library. The gels on the left contain controls with empty pUC19 plasmid without any protein added (–), with Cas12a but no crRNA (-cr) or with Cas12a-crRNA but with only the empty pUC19 plasmid (-A) or the plasmid library (library) at 10 mM $MgCl_2$ incubated at 37 °C for 30 min.

C) Cleavage of single-stranded M13 phage DNA by Cas12a activated with a short double-stranded DNA activator. The time points were 15 s, 30 min, and 60 min. The first four lanes are controls with M13 DNA without any protein added (–), M13 DNA with only Cas12a added (-cr), Cas12a-crRNA but without the activator DNA added (-A), or Cas12a-crRNA with a non-targeting dsDNA oligo added (+N). All controls contained 10 mM $MgCl_2$ and were incubated at 37 °C for the longest time point.

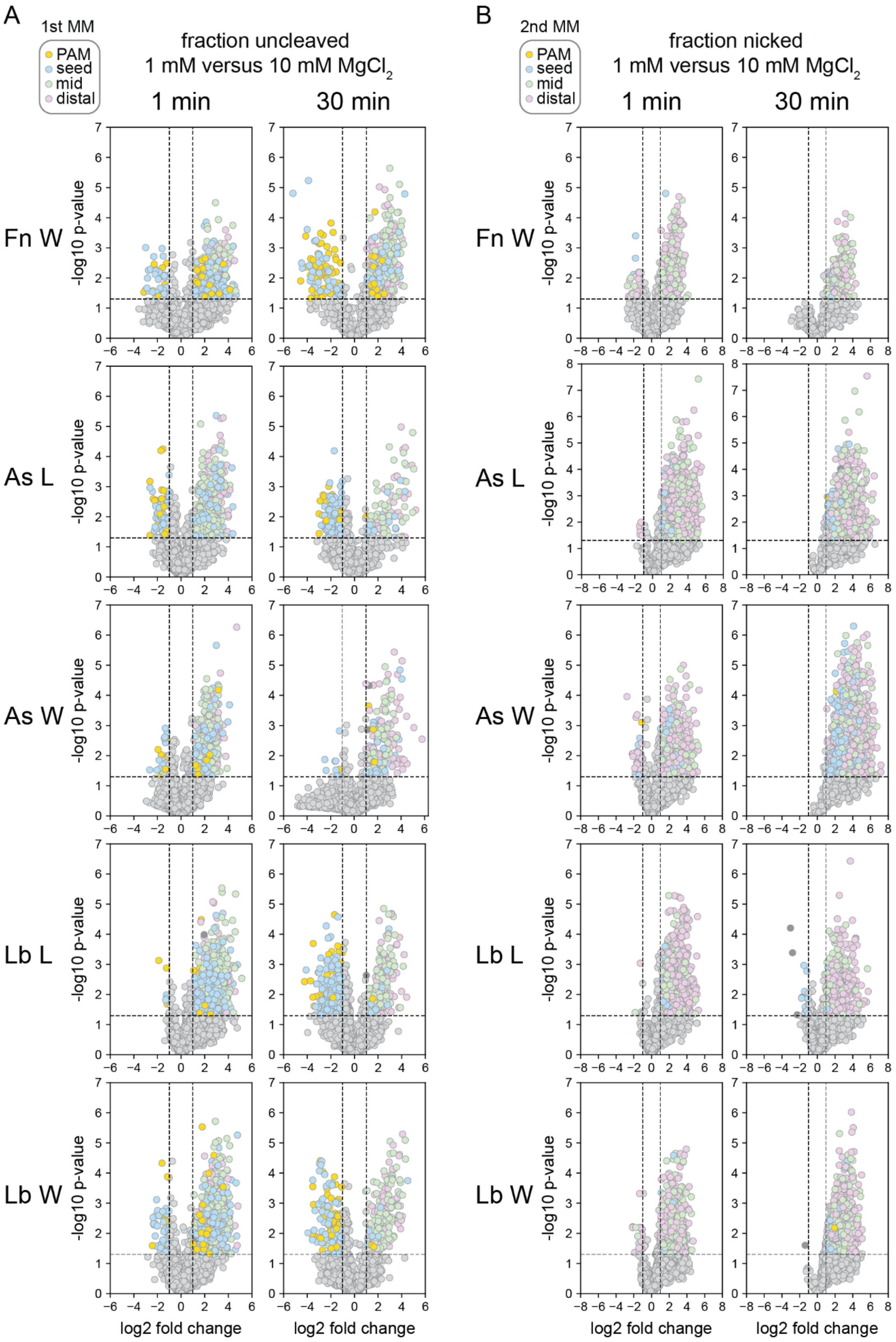

A
1st MM
PAM
seed
mid
distal
fraction uncleaved
1 mM versus 10 mM MgCl2
1 min
30 min
B
2nd MM
fraction nicked
Fn W
As L
As W
Lb L
Lb W
-log10 p-value
log2 fold change

Figure S3: Volcano plots for uncleaved and nicked fractions for all Cas12a orthologs and libraries.

(A-B) Volcano plots comparing sequences present in uncleaved (A) or nicked (B) fractions following cleavage at 1 or 10 mM $MgCl_2$ for 1 or 30 min. Data points in (A) are colored by the location of the first mutation in the sequence. The plots for each ortholog FnCas12a (Fn), AsCas12a (As) or LbCas12a (Lb) and the gene L or W library are shown. Plots for gene L cleavage by FnCas12a are shown in Fig. 2A-B. Data points in (B) are colored by the location of the second mutation in the sequence. *P* values compare up to three replicates using an unpaired two-tailed *t* test

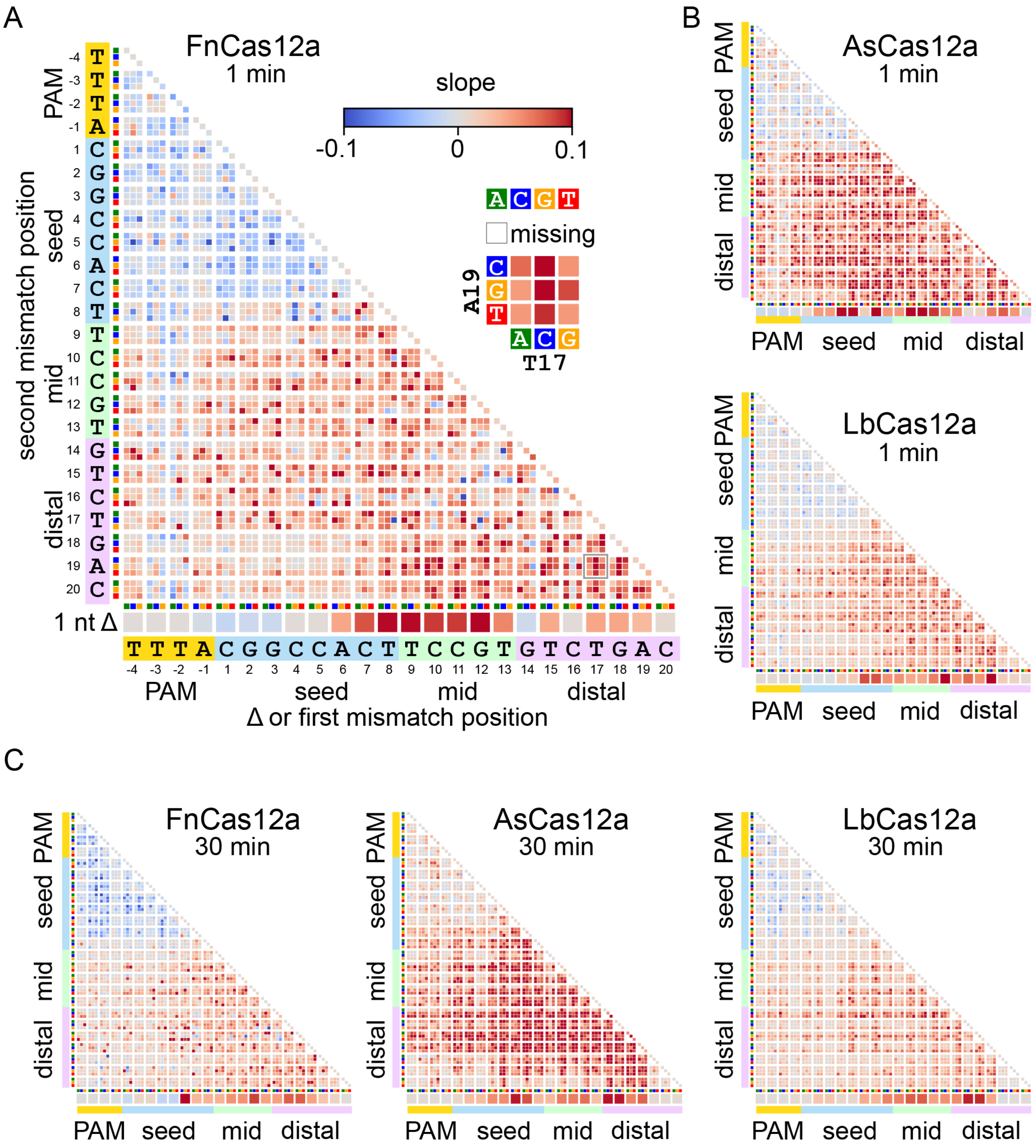

Figure S4: Slope heatmaps for the gene W target (Related to Figure 3)

A) Heatmap plotting slopes for fully cleaved DNA versus Mg^{2+} as determined in Figure 3A-B. The heatmap is for the gene W target plasmid library following 1 min cleavage by FnCas12a. Blue boxes represent sequences with a negative slope, meaning that the target was cleaved more at lower

Mg^{2+} concentration. Red boxes represent sequences with a positive slope, meaning that the target was cleaved less at lower Mg^{2+} concentration. Missing sequences are represented by white boxes.

B) Slope heatmaps of the gene W target plasmid library following 1 min cleavage by AsCas12a or LbCas12a.

C) Slope heatmaps of the gene W target plasmid library following 30 min cleavage by all three orthologs.

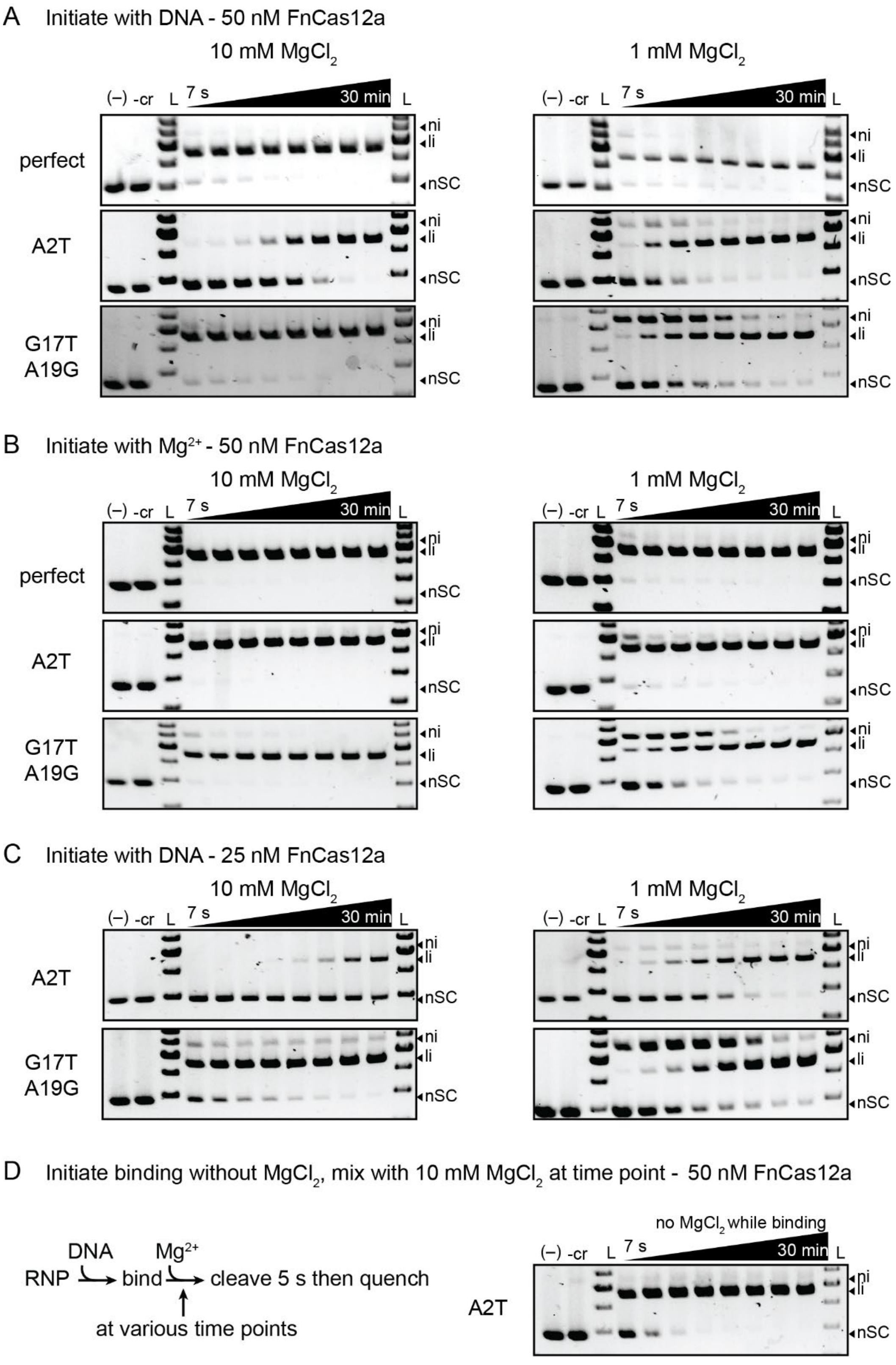
A Initiate with DNA - 50 nM FnCas12a
10 mM MgCl2
1 mM MgCl2
(–) -cr L 7 s 30 min L
perfect
A2T
G17T A19G
ni
li
nSC
B Initiate with Mg2+ - 50 nM FnCas12a
10 mM MgCl2
1 mM MgCl2
C Initiate with DNA - 25 nM FnCas12a
10 mM MgCl2
1 mM MgCl2
D Initiate binding without MgCl2, mix with 10 mM MgCl2 at time point - 50 nM FnCas12a
DNA
Mg2+
RNP
bind
cleave 5 s then quench
at various time points
no MgCl2 while binding
A2T

Figure S5: Representative agarose gels for cleavage assays quantified in Figure 4 (Related to Figure 4)

Cleavage of plasmids bearing the indicated gene L target sequence by FnCas12a for the conditions described below. The gene L sequence either perfectly matched the crRNA (perfect) or contained a seed (A2T) or two PAM-distal (G17T A19G) mutations. For all gels, (–) indicates a control in which no protein was added to the DNA and -cr indicates a control in which only Cas12a without crRNA was added to DNA before incubating at 37 °C for 30 min. Controls were performed at the same $MgCl_2$ concentration as the cleavage reaction. The time points for all gels were 7 s, 15 s, 30 s, 1 min, 2 min, 5 min, 15 min and 30 min. Each gel is a representative of at least three replicates. ni = nicked, li = linear, nSC = negatively supercoiled.

A) Gels associated with the quantified data shown in Figure 4B and 4D (50 nM RNP) and 4E (A2T mutant) in which Cas12a cleavage was initiated by mixing Cas12a-crRNA RNP together with DNA in the presence of the indicated concentration of $MgCl_2$. The gels for the perfect target are also shown in Fig. S1C.

B) Gels associated with the quantified data shown in Figure 4C in which Cas12a-crRNA RNP was first incubated with DNA for 30 min prior to addition of the indicated concentration of $MgCl_2$ to initiate cleavage.

C) Gels associated with the quantified data shown in Figure 4D (25 nM RNP) in which Cas12a cleavage was initiated by mixing Cas12a-crRNA RNP together with DNA in the presence of the indicated concentration of $MgCl_2$.

D) Gels associated with the quantified data shown in Figure 4E in which binding was initiated in the absence of $MgCl_2$. A schematic on the left describes how these reactions were performed. An RNP-DNA binding reaction was initiated in the absence of $MgCl_2$. At each time point, an aliquot from the binding reaction was mixed with 10 mM $MgCl_2$ for 5 s, followed by immediate quenching.

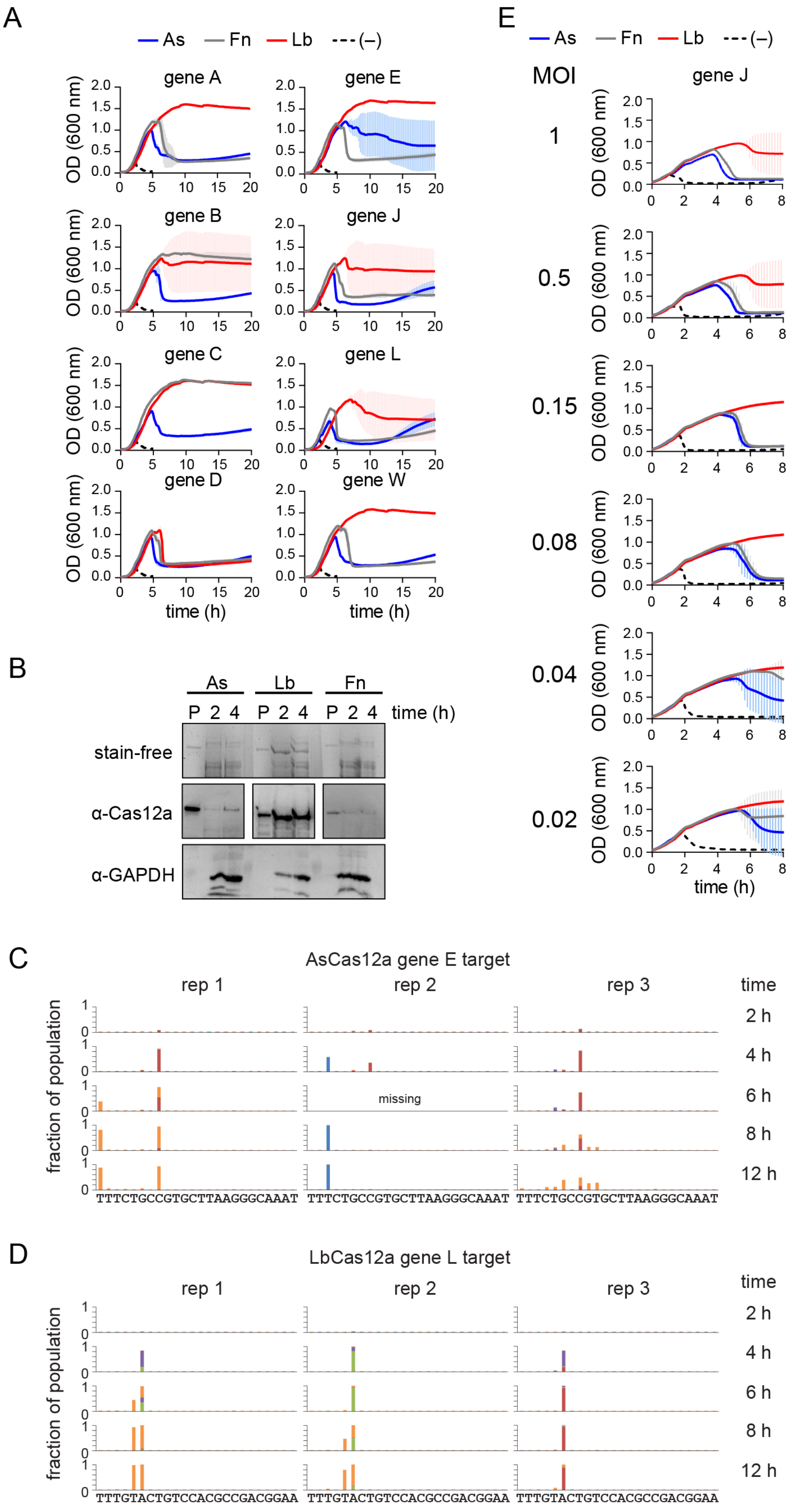
A
As Fn Lb (–)
gene A
gene E
gene B
gene J
gene C
gene L
gene D
gene W
OD (600 nm)
time (h)
E
MOI
1
0.5
0.15
0.08
0.04
0.02
B
As Lb Fn
P 2 4 P 2 4 P 2 4 time (h)
stain-free
α-Cas12a
α-GAPDH
C
AsCas12a gene E target
rep 1
rep 2
rep 3
time
2 h
4 h
6 h
8 h
12 h
missing
fraction of population
TTTCTGCCGTGCTTAAGGGCAAAT
D
LbCas12a gene L target
TTTGTACTGTCCACGCCGACGGAA

Figure S6: Growth curves, Western blots and individual replicates for phage escape experiments (Related to Figure 5)

A) Growth curves (optical density at 600 nm wavelength vs. time) for *E. coli* cultures expressing each Cas12a ortholog and a crRNA targeting the indicated gene. Cultures were infected with phage λ_{vir} at an OD_{600} of 0.3. The average of three replicates are shown and error bars represent standard deviation. Large error bars indicate that some replicates did not undergo lysis while some did.

B) Western blot analysis of Cas12a ortholog expression in *E. coli* cultures used for phage escape assays. The top panel shows the SDS-PAGE gel visualized using 2,2,2-trichloroethanol stain-free imaging. Purified AsCas12a, FnCas12a or LbCas12a (~150 ng) was loaded in the first of three lanes, followed by lysates of cultures expressing each ortholog harvested at the indicated time points. The middle panel shows the Western blots using antibodies against each individual Cas12a ortholog. The bottom panel shows a Western blot using an antibody against *E. coli* GAPDH as a loading control for the lysates.

C-D) Bar graphs showing double mutations that arose over time for three replicates of cultures expressing AsCas12a bearing a crRNA targeting gene E (C) or LbCas12a bearing a crRNA targeting gene L. A second mutation arose after an initial mutation for two of three replicates for each set of cultures.

E) Growth curves for *E. coli* cultures expressing each Cas12a ortholog and a crRNA targeting gene J infected with the indicated MOI of phage λ_{vir} at an OD_{600} of 0.3. The average of three replicates are shown and error bars represent standard deviation. Large error bars indicate that some replicates did not undergo lysis while some did.

Supplementary Data 1: Animated gifs showing the abundance of each mutant sequence in the uncleaved or nicked fraction. Each gif shows all four Mg^{2+} concentrations for a given ortholog and gene at a given time point.

Supplementary Data 2: All plasmids, primers and oligonucleotides used in this study.
